# Trait Rumination Predicts Elevated Evening Cortisol in Sexual and Gender Minority Young Adults

**DOI:** 10.3390/ijerph14111365

**Published:** 2017-11-09

**Authors:** Peggy M. Zoccola, Andrew W. Manigault, Wilson S. Figueroa, Cari Hollenbeck, Anna Mendlein, Alex Woody, Katrina Hamilton, Matt Scanlin, Ryan C. Johnson

**Affiliations:** 1 Department of Psychology, Ohio University, 200 Porter Hall, Athens, OH 45701, USA; andrewmanigaultw@gmail.com (A.W.M.); wsfigueroa@gmail.com (W.S.F.); hollenbeck.ohiou@gmail.com (C.H.); mendlein.2@buckeyemail.osu.edu (A.M.); William.Woody@osumc.edu (A.W.); kh353709@ohio.edu (K.H.); ms269111@ohio.edu (M.S.); johnsor4@ohio.edu (R.C.J.); 2 Department of Social and Public Health, Ohio University, Grover Center W324, Athens, OH 45701, USA; 3 Institute for Behavioral Medicine Research, The Ohio State University College of Medicine, 460 Medical Center Drive, Columbus, OH 43210, USA; 4 Heritage College of Osteopathic Medicine, Ohio University, 35 W. Green Drive, Athens, OH 45701, USA

**Keywords:** rumination, cortisol, stress, recovery, sexual and gender minority, depressed mood

## Abstract

Stress may contribute to illness through the impaired recovery or sustained activity of stress-responsive biological systems. Rumination, or mental rehearsal of past stressors, may alter the body’s stress-responsive systems by amplifying and prolonging exposure to physiological mediators, such as cortisol. The primary aim of the current investigation was to test the extent to which the tendency to ruminate on stress predicts diminished diurnal cortisol recovery (i.e., elevated evening cortisol) in a sample of sexual and gender minority young adults. Participants included 58 lesbian, gay, bisexual, and transgender young adults (*M*_age_ = 25.0, *SD* = 4.1) who completed an initial online survey that assessed trait rumination and current depressed mood. Participants completed daily evening questionnaires and provided salivary cortisol samples at wake, 45 min post-wake, 12 h post-wake, and at bedtime over seven consecutive days. Trait rumination predicted significantly higher cortisol concentrations at bedtime, but was unrelated to other cortisol indices (e.g., morning cortisol, diurnal slope, total output). The association with trait rumination was not accounted for by daily negative affect, and was largely independent of depressed mood. These results have implications for identifying and treating those who may be at risk for impaired diurnal cortisol recovery and associated negative health outcomes.

## 1. Introduction

As internal or external stimuli that challenge a person, stressors elicit changes in mood (e.g., increased negative affect), behavior (e.g., increased arousal and vigilance), and physiological function (e.g., increased neuroendocrine and cardiovascular reactivity [1]). Acutely, these changes can be beneficial for dealing with the stressful situation at hand, but, over time, alterations in stress-related systems can contribute to allostatic load, or detrimental wear and tear on the body [2]. For example, increased activation of the hypothalamic pituitary adrenal (HPA) axis (e.g., cortisol) and cardiovascular system (e.g., heart rate and blood pressure) collectively can improve blood flow, oxygenation, energy availability, and immune function to help an individual meet the demands of a stressor in the short term [3]. However, when the magnitude or duration of physiological stress responding is excessive (e.g., impaired recovery), it contributes to allostatic load, manifested as an increased risk for the development or progression of diseases and disorders, such as diabetes, cardiovascular disease, depression, and anxiety [4,5,6].

Critical to physiological stress responding and allostatic load is the HPA axis [3]. The HPA axis is a neuroendocrine cascade that begins in the central nervous system but ends with the release of the hormone cortisol from the adrenal glands in the body’s periphery. Broadly, cortisol assists in the regulation of metabolism, appetite, inflammation and immune functions, reproduction, cardiovascular function, and cognition. In response to physical and psychological stressors, activation of the HPA axis results in acute increases in cortisol concentrations. In addition to responding to acute threats, the HPA axis is regulated both by circadian rhythms that respond to changes in the day/night cycle of the external environment and by a negative feedback loop. As such, cortisol concentrations typically exhibit a diurnal pattern, with a sharp increase from wake to a daily peak occurring approximately 30 to 45 min after awakening and a decline to a nadir around midnight [7]. Repeated or prolonged stress may dysregulate the HPA axis, resulting in either hyper- or hypo-arousal of the HPA axis [3]. This is problematic because non-normative diurnal salivary cortisol profiles (i.e., flattened daily cortisol slopes, elevated evening cortisol concentrations) have been linked prospectively to a variety of adverse conditions, including type II diabetes, cardiovascular disease, and mortality [8,9,10,11]. Given the significant and wide-ranging consequences and correlates of HPA axis activation, it is important to document for whom and under what circumstances cortisol concentrations are altered in response to stressors and in daily life.

Cognitive processes such as rumination—repetitive past-oriented thought about negative content (e.g., stressful events, negative mood) [12,13]—can enhance or prolong physiological stress responses and exacerbate somatic symptoms [14]. For example, individuals with a tendency to ruminate exhibited greater cortisol reactivity and delayed recovery in response to a laboratory-based psychosocial stressor [15]. Although rumination is an emotion regulation process that is fairly stable over time [16], there is also within-person variability, such that some situations or emotions elicit greater ruminative thought than others (e.g., [17]). In the present study, trait rumination is defined as the tendency to mentally dwell upon, or cognitively rehearse, past stressful experiences. It is important to note that the present conceptualization of trait stress-related rumination is distinct from depressive rumination, which reflects dwelling on symptoms of depression (e.g., sadness) and the consequences of such symptoms and has been identified as a major risk factor for the development and progression of depression (e.g., [18]). As past work has shown, depressive rumination predicts different cortisol stress responses than stress-related rumination does (for review, see [19]).

To date, a handful of studies have documented the association between trait stress-related rumination and diurnal or evening cortisol. For example, in our past work, we have found that among university students, individuals with high scores on a measure of trait stress-related rumination did not exhibit the expected drop in cortisol concentrations on the evening following a laboratory speech stressor [20]. This suggests that ruminating on a stressful circumstance may prevent the normative diurnal cortisol decline. Similarly, two other studies of working adults found that trait stress-related rumination [21] and work-related rumination [22] predicted elevated evening cortisol. In the latter study, rumination was also related to a flattened cortisol response to awakening.

Although our focus is on stress-related rumination, it may be informative to examine other studies that have used standard or modified depressive rumination measures to investigate the relationship between ruminative thought and cortisol. In one such study, trait depressive self-focused rumination was associated with lower cortisol awakening responses, but not total diurnal cortisol output (calculated as area under the curve with respect to ground; AUCg) assessed across one day in a sample of undergraduate students [23]. In another study of early adolescent girls, a three-item modified trait depressive rumination measure focused on peer problems was related to lower waking cortisol and flatter diurnal slope, but no other cortisol parameter assessed over three consecutive weekdays [24]. In a third study, trait depressive rumination in remitted depressed patients and healthy controls was not related to cortisol concentrations assessed five times per day over two days [25]; however, mean daily ruminative self-focus (i.e., thinking about my problems and my feelings) was associated with elevated cortisol concentrations in patients and controls.

Taken together, these initial findings suggest that trait stress-related rumination may be related to elevated diurnal cortisol—particularly in the evening. However, some of the past work has limitations, including using only one day or just a few days of cortisol assessment and not evaluating the independent effects of stress-related rumination and depressed mood. As such, the current study sought to overcome some prior methodological challenges by examining diurnal cortisol over the course of a full week and taking depressed mood and negative affect into consideration.

In addition, the current project focused on lesbian, gay, bisexual, and transgender (LGBT) young adults as a population of interest to replicate and extend past work. Theoretical models of stress and health suggest that LGBT individuals experience specific minority stressors (i.e., external stressors like overt discrimination and internal stressors such as internalization of negative stereotypes) that can directly and indirectly influence health outcomes [26]. Indirect influences on health may operate via health behaviors (e.g., smoking as way cope with stress) or physiological processes (e.g., greater exposure to stress hormones like cortisol). Past meta-analytic work has shown that minority stressors may lead to dysregulated physiological responses which, in turn, can lead to poorer health [27]. For example, the experience of recent LGBT-related stressful life events (e.g., arguments with friends or family over homosexuality/bisexuality) was related to flatter diurnal cortisol slope, and, in turn, more depressive symptoms in a sample of sexual minority young adults [28]. Moreover, although the diurnal cortisol levels of sexual minority individuals may not differ from their heterosexual counterparts (although note that transgender individuals were not included) [29], LGBT adolescents and young adults may be especially vulnerable to minority stress, as self-identification and identity disclosures are commonly made during this developmental period [30]. Furthermore, stigma and shame-related processes are potent elicitors of HPA axis activity [20,31,32] and rumination [17,33,34,35]. Finally, we focused on LGBT adults from relatively rural and small-town locations in the Midwest of the United States as they may be at greater risk for minority stress and associated health outcomes, relative to their counterparts in major metropolitan areas [36].

The primary aim of the current investigation was to test the extent to which trait stress-related rumination predicts diminished diurnal cortisol recovery in a sample of LGBT young adults. Based on prior literature, we expected to find that individuals with greater tendencies to ruminate would have elevated evening salivary cortisol. We also explored whether trait rumination was related to greater overall daily cortisol output (AUCg) and diurnal cortisol trajectories (slope). Given the extant links between rumination and depression, we also tested the extent to which any rumination–cortisol associations are independent of depressed mood and negative affect in daily life.

## 2. Materials and Methods

### 2.1. Participants

A flexible recruitment approach was used to identify LGBT individuals for a larger project examining the stress and health of sexual and gender minority young adults. We advertised with a variety of organizations and utilized snowball sampling methods that have been successfully applied in past work to recruit difficult-to-reach samples, including sexual minorities (e.g., [37,38]). Consistent with these approaches, we used e-mail and online communications to target university- and community-based LGBT organizations and listservs based in Ohio and the surrounding Midwestern region of the United States. Study recruitment materials were also posted on relevant social media sites (e.g., public LGBT groups on Facebook). In addition, individuals were asked to reach out to friends and acquaintances who they thought may be eligible for the study. Participants were considered eligible if they were between 18 and 35 years of age, identified as a sexual or gender minority (i.e., not both heterosexual and cisgender), had reliable access to the Internet between the hours of 9:00 PM and 1:00 AM, were not pregnant, and did not use steroid medication or report a major psychiatric/endocrine disorder. A total of 121 individuals enrolled in the full study. Among these participants, a randomly selected subset (*n* = 58) was asked to provide four saliva samples per day during the week of data collection. The current sample was limited to those with useable cortisol data (*n* = 49); four participants did not follow saliva sampling procedures, and five did not return saliva samples (88.6% completion rate). The final sample included 49 individuals who were, on average, 24.8 years of age (*SD* = 4.1 years, range = 18–34). Of the 49, 30 participants reported female biological sex at birth and 19 reported male sex at birth. The sample was predominantly White (79% White; 14% multi-racial, and 6% other race), university-educated (61% with at least a college degree), employed (61% full-time, 29% part-time, 10% not employed), homosexual (61% homosexual; 21% bisexual, and 18% other sexuality), and cisgender (82% cisgender; 10% transgender, and 8% unsure).

### 2.2. Procedure

All participants completed an initial online screening survey that was linked to recruitment communications. At completion of the screening, eligible participants were immediately invited to complete the week-long study of “Daily Activities, Stress, & Health”. Interested individuals provided consent online and then completed an initial survey which included a variety of questionnaires regarding health behaviors, trait measures, and sociodemographics. Those who fully completed the initial survey were invited into the week-long portion of the study. Participants were randomly assigned to one of two arms of the larger study: (1) complete evening surveys only (*n* = 63); or (2) complete evening surveys plus cortisol sampling (*n* = 58). Informed consent for this part of the study was obtained over the phone and in writing via a paper version that was returned when the participant had completed the study. For seven consecutive days, participants were asked to collect saliva four times per day and complete evening online surveys lasting 15–20 min. To minimize variation in reporting times, participants were only able to complete the evening survey between 9:00 PM and 1:00 AM (participants’ local time). Individuals participating in the cortisol sampling procedure were mailed pre-packaged saliva collection kits that contained time-labeled, color-coded cryogenic saliva storage vials, two-inch straws to collect saliva, illustrated saliva collection instructions, paper logs (to assess sampling time, wake time, bedtime, food intake, physical activity, and other daily activities), and pre-paid return mailers. To aid compliance, participants were sent reminders (in the form of text messages and e-mails), and urged to set their own alarms. Participants were compensated $15 for completing the initial survey, $5 for completing each daily evening survey, $5 for each day of completed saliva collection, a $5 bonus for completing all evening surveys, and a $5 bonus for completing all saliva samples. In total, participants could receive up to $95 for full participation. Data were collected between April 2015 and July 2016. The investigations were carried out following the rules of the Declaration of Helsinki, and the protocol was approved by the Ohio University Institutional Review Board (14F057).

### 2.3. Measures

#### 2.3.1. Salivary Cortisol

For seven consecutive days, participants were instructed to collect saliva upon awakening, 45 min post-wake, 12 h post-wake, and immediately before going to bed. Number of days and sampling times were guided by the MacArthur Research Network recommendations [39]. The passive drool method was used to collect saliva samples in four sterile 2.0 mL cryogenic storage vials. Participants were asked to store samples in a freezer before mailing them back to the laboratory. Salivary cortisol samples stored at 5 °C do not degrade for up to 3 months [40] and concentrations are stable across extended periods of time at room temperature (i.e., beyond the typical mailing time of 2–5 days [41]). All returned samples arrived at the laboratory within 3 months of collection. Upon arrival to the research facility, samples were stored at −80 °C until centrifuged and assayed in duplicate (results averaged) using enzyme-linked immunosorbent assays (R&D Systems, Inc., Minneapolis, MN, USA) at Ohio University. The lower limit of detection for assays was 0.07 ng/mL; intra- and inter-assay variation coefficients of variation were less than 10%. To address positive skewness, salivary cortisol values were natural log transformed.

#### 2.3.2. Trait Rumination

The 14 item Rehearsal subscale of the Revised Emotion Control Questionnaire was used to assess trait stress-related rumination [42]. Participants rated the degree to which a given statement (e.g., “I find it hard to get thoughts about things that have upset me out of my mind.”) was “most like” them by selecting either “1 = True” or “0 = False”. Scores on this scale were summed (*M* = 6.10, *SD* = 3.30, α = 0.78).

#### 2.3.3. Depressed Mood

Depressed mood over the past week was assessed during the initial survey with the 10 item version of the Center for Epidemiologic Studies Depression Scale [43]. Participants rated how often a given statement (e.g., “I felt depressed.”) occurred over the past week on a four-point scale (0 = Rarely or none of the time (less than 1 day); 3 = Most or all of the time (5–7 days)). Scores were summed (*M* = 10.83, *SD* = 5.40, α = 0.81).

#### 2.3.4. Daily Negative Affect

Negative affect was measured using the 10 item subscale of the Positive Affect Negative Affect Scale [44]. As part of daily surveys, participants rated the degree to which they had “felt this way” (e.g., afraid, nervous, upset) on a five-point scale (1 = not at all; 5 = a great deal) on the day of the survey. Scores were averaged for each day separately (*M*_week_ = 1.46, *SD*_week_ = 0.58, αs range = 0.80 to 0.94).

#### 2.3.5. Additional Descriptive Variables and Covariates

A variety of factors that may influence diurnal cortisol or be associated with trait rumination were assessed with the initial survey, evening surveys, or with the saliva sampling logs. Such variables included time of awakening, bedtime, day of the week, and biological sex at birth [45]. Day of the week was coded as a binary variable (1 = weekend: Saturday and Sunday; 0 = weekday), while self-reported time of wake (*M* = 8.43, *SD* = 1.76) and bedtime (*M* = 24.08, *SD* = 1.48) were recoded as hours since midnight.

### 2.4. Statistical Analyses

Analyses predicting diurnal cortisol values were performed with multilevel modeling using the SAS 9.4 PROC MIXED procedure (SAS Institute, Cary, NC, USA). Diurnal cortisol trajectories and bedtime cortisol levels were modeled using three-level models, in which samples were nested within days and days were nested within individuals. For analyses predicting diurnal cortisol trajectories, we modeled temporal changes in cortisol as a cubic function of time (in hours, centered at wake) because it provided the best fit to the data (i.e., two inflection points in the diurnal cortisol trajectory). Trait rumination, depressed mood, day-to-day negative affect, and covariates were all allowed to interact with cubic diurnal cortisol slopes in the three-level models. Day-level and individual-level intercepts were modeled as random effects using unstructured covariance matrices. Similarly, day-level and individual-level linear slopes were modeled as random effects using unstructured covariance matrices. To quantify daily cortisol output, area under the curve with respect to ground (AUCg) was computed for each day using the trapezoidal method [46]. Computing AUCg using the trapezoidal method requires all four cortisol values and sampling time data for a given day. For this reason, AUCg could only be computed for 82% of days during which cortisol was sampled. Cortisol AUCg and self-reported bedtimes were modeled using two-level models, in which days were nested within participants and day-level intercepts were allowed to vary randomly. Maximum likelihood mixed models were used to analyze cortisol AUCg, diurnal cortisol trajectories, and bedtime cortisol levels, as well as self-reported bedtimes.

Outlying cortisol sampling times (±3 *SDs* from instructed sampling times or the average bedtime (15.5 h post-wake, ±2.00 h)) were excluded from final analyses predicting cortisol levels (*n* = 21, 1.59%), and cortisol values exceeding 60 nmol/L were considered implausibly high [47] and removed (*n* = 15, 1.14%), yielding a final sample of 1278 cortisol values. Self-reported bedtime data was available for 91.5% of days (7 outlying values; 21 missing values). All statistical tests were two-tailed with a significance level set at *p* < 0.05 and borderline significance set at *p* < 0.10.

## 3. Results

### 3.1. Descriptive Results

Men and women had equivalent levels of trait rumination and depressed mood, and neither trait rumination nor depressed mood differed by sexual orientation (all *p*s > 0.05). There was a moderate positive correlation between trait rumination and depressed mood, with values *r*(49) = 0.44 and *p* = 0.002. Correlations between trait rumination and daily negative affect measures were positive and ranged from *r*(49) = 0.19, *p* = 0.069 to *r*(49) = 0.35, *p* = 0.001.

Diurnal cortisol levels fluctuated across the day as a cubic function of time (*β* = 0.0019, *F*(1, 530) = 28.13, *p* < 0.001), such that cortisol concentrations rose from wake to 45 min post-wake, decreased from 45 min post-wake to 12 h post-wake, and continued to decrease at a slower rate from 12 h post-wake to bedtime.

As shown in Table 1, multiple variables were related to cortisol indices. For example, diurnal cortisol output (AUCg) was lower on days that participants awoke later. Wake time also interacted with cubic diurnal cortisol slopes (*β* = −0.0005, *F*(1, 571) = 6.94, *p* = 0.008). Follow-up analyses revealed that later wake times were associated with lower cortisol levels 45 min post-wake (*β* = −0.0788, *F*(1, 571) = 10.39, *p* = 0.001) and 12 h post-wake (*β* = −0.0762, *F*(1, 570) = 6.89, *p* = 0.008). Diurnal cortisol output was greater on weekdays than weekends, and cortisol levels were significantly larger on weekdays relative to weekends 45 min post-wake and marginally greater on weekdays relative to weekends 12 h post-wake. Self-reported bedtime and biological sex did not predict cortisol AUCg, diurnal cortisol slopes, or individual cortisol intercepts (all *p*s > 0.05), and thus were not retained as covariates in subsequent analyses.

### 3.2. Rumination and Cortisol Results

**Figure 1:**
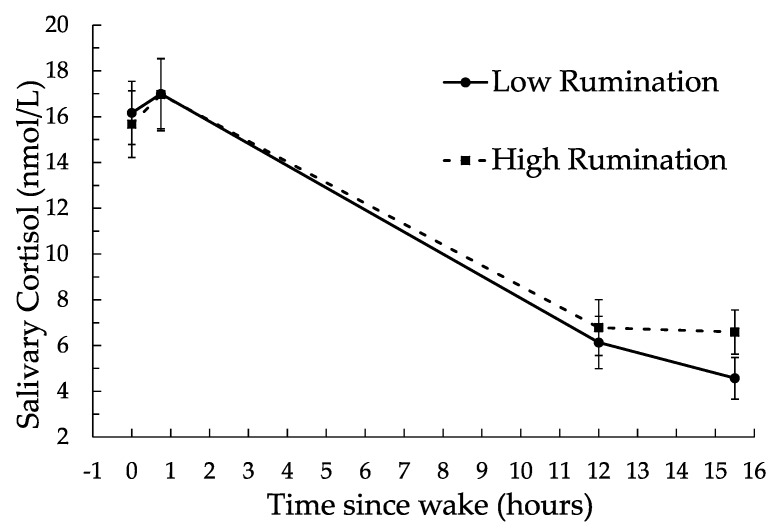
Diurnal salivary cortisol as predicted by trait rumination. Data points shown are estimated means (along with standard errors of mean estimates) for high (+1 *SD*) and low (−1 *SD*) levels of rumination. Precise sampling time guidelines were provided for samples taken at wake, 45 min post-wake, 12 h post-wake, and at bedtime (*M* = 15.5 h post-wake).

The relationship between trait rumination and diurnal cortisol is depicted in Figure 1. As shown, trait rumination predicted significantly higher cortisol concentrations at bedtime (*β* = 0.0774, *F*(1, 527) = 8.07, *p* = 0.004), controlling for waketime and day of the week. Trait rumination was not related to cortisol concentrations at wake, 45 min post-wake, or 12 h post-wake, with values *β* = 0.0120, *F*(1, 522) = 0.33, *p* = 0.56; *β* = 0.0011, *F*(1, 522) = 0.00, *p* = 0.95; and *β* = 0.0363, *F*(1, 522) = 2.52, *p* = 0.11; respectively. Trait rumination was also unrelated to diurnal cortisol output or diurnal cortisol slopes, with values *β* = 0.0147, *F*(1, 208) = 0.53, *p* = 0.46; and *β* = −0.00006, *F*(1, 522) = 0.28, *p* = 0.59; respectively.

### 3.3. Depressed Mood and Daily Negative Affect

Depressed mood did not predict cortisol output (AUCg, *β* = −0.003, *F*(1, 209) = 0.06, *p* = 0.79), or interact with cubic diurnal cortisol slopes (*β* = −0.0001, *F*(1, 523) = 2.33, *p* = 0.12), controlling for waketime and day of the week. Follow-up analyses revealed that there was a significant association between greater depressed mood and greater bedtime cortisol levels (*β* = 0.041, *F*(1, 523) = 5.20, *p* = 0.023). When controlling for depressed mood, the association between trait rumination and bedtime cortisol levels was marginally significant (*β* = 0.053, *F*(1, 521) = 3.03, *p* = 0.082).

On days that more negative affect was reported, cortisol levels 12 h post-wake were significantly greater (*β* = 0.24, *F*(1, 522) = 5.98, *p* = 0.014), controlling for waketime and day of the week. Daily reports of negative affect were not related to other cortisol indices (all *p*s > 0.05). The association between trait rumination and bedtime cortisol levels remained significant after controlling for daily negative affect (*β* = 0.066, *F*(1, 521) = 5.95, *p* = 0.015).

### 3.4. Additional Exploratory Results

The relationship between trait rumination and bedtime cortisol levels was not moderated by sex, day of the week, or self-reported bedtime (all *p*s > 0.05). In addition, trait rumination did not interact with depressed mood or negative affect to predict evening cortisol (all *p*s > 0.05). Further, the association between trait rumination and bedtime cortisol levels remained marginally significant after concurrently controlling for daily negative affect and depressed mood as well as waketime and day of the week (*β* = 0.051, *F*(1, 518) = 2.82, *p* = 0.093). Finally, trait rumination did not predict the time at which participants went to bed (*β* = −0.0248, *F*(1, 267) = 0.52, *p* = 0.46).

## 4. Discussion

Rumination is considered to be a psychological mechanism that may contribute to stress-related disease processes resulting from prolonged activation or impaired recovery of stress-related biological systems [14]. Although there is growing support for this premise [48], prior research focused on rumination and diurnal cortisol in daily life has been somewhat mixed. The present study examined the association between trait stress-related rumination and diurnal cortisol assessed over the course of a week in a sample of LGBT young adults. Our main hypothesis was that trait rumination would predict elevated evening cortisol (i.e., impaired diurnal cortisol recovery).

As predicted, we found that greater trait rumination scores were related to greater evening cortisol across the week-long assessment period. This finding is consistent with two past studies, in which greater trait stress-related rumination [21] and work-related rumination [22] predicted elevated evening cortisol among working adults, as well as an additional study that linked trait stress-related rumination to elevated cortisol on the evening following a laboratory speech stressor [20]. Our results also suggest that rumination may prevent the normative evening decline in diurnal cortisol, or impaired diurnal cortisol rhythm, which has important health implications. Multiple studies have documented prospective links between increased evening cortisol concentrations and disease incidence and mortality. For example, elevated evening cortisol predicted new-onset type II diabetes and impaired fasting glucose in the subsequent nine-year follow-up period [11]. In another study, flattened slope, with raised evening cortisol in particular, predicted an increase in all-cause and cardiovascular mortality six years later [8].

We also explored whether trait rumination would be associated with other cortisol indices, including total output (AUCg), diurnal slope, and other intercepts (e.g., waking cortisol), as some prior work has connected other forms of ruminative thought to these cortisol measures (e.g., [23,24,25]). For example, trait self-focused depressive rumination has been linked to a lower cortisol awakening response [23], and depressive rumination on peer problems was tied to lower waking cortisol concentrations in a sample of adolescents [24]. In the current study, we found no associations between trait stress-related rumination and other cortisol measures. These seemingly discrepant findings may be due in part to how rumination was assessed. Although stress-related and depression-related rumination may share some similar features (i.e., repetitive focus on negative content), the two are conceptually distinct and have been differentially linked to cortisol reactivity to acute stress in past work (i.e., trait depressive rumination was associated with blunted cortisol reactivity whereas state stress-related rumination was related to enhanced cortisol reactivity [49]). It is of note that neither the present nor past studies linked rumination to total cortisol output (AUCg), which does not capture the important temporal dynamics of diurnal cortisol concentrations.

Temporal dynamics are important to consider in understanding diurnal rumination and cortisol patterns. Some research points to a diurnal pattern of unpleasant mood and cognition, and that rumination may be more likely to occur in the evening [50]. In addition, a week-long experience sampling study of rumination and negative affect demonstrated that ruminative self-focus was highest in the morning and evening, perhaps reflecting anticipating the day ahead or reviewing the day prior during relatively distraction-free times of the day [51]. Taken together, it is perhaps not surprising to find an association between trait rumination and evening cortisol, but no links to other cortisol indices in the current study. Diurnal influences on rumination and cortisol patterns warrant additional attention in future studies.

Because there are well-documented links between ruminative thought, depressed mood, and negative affect (e.g., [51]), we also explored the extent to which the rumination–cortisol association was independent of depressed mood and daily negative affect. In the current study, we report small to moderate correlations between depressed mood, daily negative affect, and trait rumination. When we re-examined the association between trait rumination and evening cortisol with depressed mood and daily negative affect, the results were largely the same; the rumination–cortisol association was independent of negative affect and the association became marginally significant with the addition of depressed mood (which had a significant independent effect on evening cortisol). It is worth noting that while we only enrolled individuals who reported no major psychiatric or medical conditions, the current sample had relatively high symptoms of depressed mood (i.e., mean levels near the screening cut-off score for identifying those at risk for depression [52]). Elevated depressive symptoms does not appear to be specific to the sexual and gender minority status of our sample, as we have found mean depressed mood levels near the established cut-off in our other studies of university students (not selected for sexual orientation) [20]. It may be informative to compare individuals with and without depression in future work to see if the relationship between rumination and evening cortisol holds across groups.

The current study focused on rumination as a dispositional construct, rather than a state process. Nonetheless, our results have implications for targeting state ruminative thinking, which is greater among trait ruminators (e.g., [51]). A variety of potential interventions could be considered in altering or reducing ruminative thought and facilitating recovery in daily life. For instance, distraction or non-reactive forms of thought (i.e., reflection) predict enhanced physiological recovery from acute stress [53,54,55]. In addition, recent meta-analytic findings indicate that cognitive behavioral interventions and mindfulness-based interventions may be effective in altering and reducing maladaptive ruminative thinking [56]. Possible next steps are to test the effects of such interventions on ruminative thinking and diurnal cortisol in daily life.

The current study is the first to report on the links between stress-related rumination and diurnal cortisol in a sample of sexual and gender minorities. Previous studies have reported on the associations between exposure to minority stress and cortisol in LGBT adults with somewhat inconsistent outcomes. For instance, some studies have linked greater minority stress exposure (e.g., arguments with friends or family over homosexuality/bisexuality, coming-out stress, transitioning-identity stress) to elevated or dysregulated diurnal cortisol in sexual and gender minority men [28,57]. However, in another study, an opposite result was found: greater disclosure of sexual orientation at the workplace (i.e., less concealment of sexual identity, which is considered a minority stressor [26]), was associated with greater cortisol output across the day in a sample of gay and bisexual men [58]. Together, these studies indicate that more work needs to be done to better understand which minority stressors or under what circumstances minority stressors are linked to cortisol. To that end, the current study shows that, beyond exposure to minority stressors, cognitive-behavioral responses to stressors (i.e., stress-related rumination) are important for understanding cortisol and minority stress processes in the LGBT community. As such, future investigations of minority stress and biological stress markers may benefit from including measures of stress-related ruminative thinking.

The vast majority of prior work on stress and health in LGBT adults has focused on samples drawn from major metropolitan areas, where LGBT resources are more plentiful and attitudes more favorable to sexual and gender minorities [36]. In the current study, we deliberately focused on the understudied population of LGBT adults from relatively rural and small-town locations because they may be at increased risk for minority stress and associated negative health outcomes. Without the inclusion of a heterosexual cisgender comparison group in the current study and without comparable published reports on rumination and diurnal cortisol in heterosexual cisgender samples (or samples unselected for sexual and gender identity), it remains unknown whether the rumination–cortisol connection is more strongly coupled among LGBT individuals relative to others. Future studies that include LGBT and non-LGBT adults and test the potential moderating role of sexual or gender identity in the rumination–cortisol association would shed light on this issue.

Although we did not include a comparison group in the current study, it should be noted that our sample did not exhibit greater rumination tendencies [20] or abnormal diurnal cortisol profiles relative to other adult samples from the United States (e.g., [59]). However, bedtime salivary cortisol values in the current sample appear to be somewhat greater. Other lines of work show that throughout their lives LGBT persons are at increased risk for many mental and physical health conditions [60,61]. For example, lesbians and bisexual men and women are at elevated risk for cardiovascular disease [62,63]. Consistent with minority stress theory [26], these health disparities may be explained in part by alterations in stress processes, such as elevated evening cortisol, which may indicate poor recovery from stress. As already discussed, separate lines of work indicate that elevated evening cortisol is linked to diabetes risk [11] and cardiovascular mortality [8]. Thus, the current findings may help to shed light on the extant health disparities between sexual and gender minorities and their heterosexual and cisgender counterparts.

The present study had several key strengths, including a full week of daily life assessments with four saliva samples collected per day, which increases the reliability of our cortisol indices relative to past work [64]. In addition, we included measures of depressed mood and daily negative affect to better tease apart stress-related rumination measure from associated depression-related constructs. Despite these strengths, the current study is not without limitations. For instance, the current study is cross-sectional, which only allows for a snapshot in time. It will be important for future research to test the prospective, longitudinal relationships between rumination, diurnal cortisol, and health outcomes. In addition, to determine the extent to which rumination predicts elevated evening cortisol across the lifespan and in different cultures, replication is warranted with samples drawn from a wider range of ages as well as with samples that are more ethnically and culturally diverse.

## 5. Conclusions

Trait rumination predicted significantly higher cortisol concentrations at bedtime, but was not related to other diurnal cortisol indices. Furthermore, this association was independent of daily negative affect and was similar when controlling for depressed mood. Given the documented links between elevated evening cortisol and future disease and mortality [8,11], these results have implications for identifying and treating those at risk for impaired diurnal cortisol recovery and associated negative health outcomes.

## Figures and Tables

**Table 1:** Salivary cortisol indices as predicted by rumination and other variables of interest.

Variables of Interest	Waking		45 min Post-Wake		12 h Post-Wake		Before Bed		AUCg	
	Estimate	SE	Estimate	SE	Estimate	SE	Estimate	SE	Estimate	SE
Intercept	2.52 **	0.06	2.67 **	0.06	1.30 **	0.08	1.00 **	0.09	4.99 **	0.06
Rumination	0.00	0.02	0.00	0.02	0.03	0.02	0.07 *	0.02	0.01	0.02
Depressed mood	0.01	0.01	−0.01	0.01	0.00	0.01	0.03 ^†^	0.02	−0.01	0.01
Negative affect	0.06	0.07	0.03	0.07	0.27 **	0.10	0.16	0.10	0.12	0.07
Wake time	0.01	0.02	−0.07 **	0.02	−0.07 **	0.02	0.01	0.03	−0.10 **	0.02
Day of the week	−0.14	0.08	−0.25 **	0.08	−0.18 ^†^	0.10	0.12	0.11	−0.21	0.07
Sex	0.12	0.13	0.13	0.12	−0.02	0.16	−0.17	0.20	0.07	0.13
Bedtime	0.01	0.02	−0.00	0.02	0.00	0.02	0.03	0.03	0.01	0.03

Notes: Estimates depict the unadjusted relationship between variables of interest and log-transformed cortisol indices. Day of the week was coded as follows: 0 = weekday; 1 = weekend. Sex represents biological sex at birth and was coded as follows: 0 = male; 1 = female. Wake time refers to self-reported wake time in hours since midnight. Bedtime refers to self-reported bedtime in hours since midnight. ^†^ *p* < 0.10; * *p* < 0.05; ** *p* < 0.01.
